# Fast Approximations of Activation Functions in Deep Neural Networks when using Posit Arithmetic

**DOI:** 10.3390/s20051515

**Published:** 2020-03-10

**Authors:** Marco Cococcioni, Federico Rossi, Emanuele Ruffaldi, Sergio Saponara

**Affiliations:** 1 Department of Information Engineering, Università di Pisa, Via Girolamo Caruso, 16, 56122 Pisa PI, Italy; marco.cococcioni@unipi.it (M.C.); federico.rossi@ing.unipi.it (F.R.); 2 Medical Microinstruments (MMI) S.p.A., Via Sterpulino, 3, 56121 Pisa PI, Italy; emanuele.ruffaldi@mmimicro.com

**Keywords:** alternative representations to float numbers, posit arithmetic, Deep Neural Networks (DNNs), neural network activation functions

## Abstract

With increasing real-time constraints being put on the use of Deep Neural Networks (DNNs) by real-time scenarios, there is the need to review information representation. A very challenging path is to employ an encoding that allows a fast processing and hardware-friendly representation of information. Among the proposed alternatives to the IEEE 754 standard regarding floating point representation of real numbers, the recently introduced Posit format has been theoretically proven to be really promising in satisfying the mentioned requirements. However, with the absence of proper hardware support for this novel type, this evaluation can be conducted only through a software emulation. While waiting for the widespread availability of the Posit Processing Units (the equivalent of the Floating Point Unit (FPU)), we can already exploit the Posit representation and the currently available Arithmetic-Logic Unit (ALU) to speed up DNNs by manipulating the low-level bit string representations of Posits. As a first step, in this paper, we present new arithmetic properties of the Posit number system with a focus on the configuration with 0 exponent bits. In particular, we propose a new class of Posit operators called L1 operators, which consists of fast and approximated versions of existing arithmetic operations or functions (e.g., hyperbolic tangent (TANH) and extended linear unit (ELU)) only using integer arithmetic. These operators introduce very interesting properties and results: (i) faster evaluation than the exact counterpart with a negligible accuracy degradation; (ii) an efficient ALU emulation of a number of Posits operations; and (iii) the possibility to vectorize operations in Posits, using existing ALU vectorized operations (such as the scalable vector extension of ARM CPUs or advanced vector extensions on Intel CPUs). As a second step, we test the proposed activation function on Posit-based DNNs, showing how 16-bit down to 10-bit Posits represent an exact replacement for 32-bit floats while 8-bit Posits could be an interesting alternative to 32-bit floats since their performances are a bit lower but their high speed and low storage properties are very appealing (leading to a lower bandwidth demand and more cache-friendly code). Finally, we point out how small Posits (i.e., up to 14 bits long) are very interesting while PPUs become widespread, since Posit operations can be tabulated in a very efficient way (see details in the text).

## 1. Introduction

Due to the pervasivenss of real-time and critical systems like Internet of Things (IoT) platforms, automotives, and robotics, new types of requirements are being addressed in the use of Deep Neural Networks (DNNs).

The main challenges when dealing with DNNs are both the ubiquitous multiply-and-accumulate operations and the massive use of activation functions across the neural network layers. A big speed-up to these challenges is surely offered by parallelization of the workloads (e.g., Graphics Processing Units (GPUs) or Single-Instruction Multiple-Data (SIMD)) processors). However, these solutions are considerable demanding in terms of resources. Moreover, adding parallelization in critical systems may reduce the predictability of the said system (see References [1,2]). Furthermore, even the use of floating point SIMD engines is not always possible in embedded systems (e.g., ARM Cortex-M4 [3]). This means that we cannot always rely on high-performance processing units in critical and real time scenarios, thus needing to address new challenges.

Therefore, the challenging topic is to satisfy the real-time requirements while guaranteeing computational efficiency and lowering the power and the cost of such applications. One of the main paths to reduce the computational complexity when evaluating DNNs is stepping away from cumbersome arithmetic such as double-precision floats (represented on 64 bit). The basic idea is to use compressed formats that may save resources in terms of power consumption and computational efficiency. Great examples of compact formats are Brain Floats (BFLOAT) and Flexpoint [4,5] that consist in an optimized version of the 16-bit standard floating point number IEEE 754) used by Google for their TPU (tensor processing unit) engines. Other formats also come from the concept of transprecision computing [6,7] (NVIDIA Turing architectures allow computation with 4-, 8-, and 32-bit integers and with 16- and 32-bit floats). The up-and-coming Posit format has been theoretically [8,9,10] and practically [11] proven to be a perfect replacement for IEEE float numbers when applied to DNNs in terms of efficiency and accuracy.

Due to its novelty, this format lacks proper or standardized hardware support (e.g., a Posit Processing Unit (PPU)) to accelerate its computation, forcing the use of software implementations. However, in order to speed up the software emulation of Posits in DNNs, we present two different techniques. In this paper, we extend the work on deriving a fast and approximate version of the hyperbolic tangent (TANH) presented in Reference [12]. We introduce novel arithmetic properties of the Posit number system with a deep focus on the Posits with 0 exponent bits. This special case allows us to build common functions and arithmetic operators as simple bit manipulations on the bit-string representing a Posit number. This new class of functions (called L1 functions) has some interesting properties:

- The operation evaluation is faster than its counterpart with little to no degradation on accuracy.
- Since these operations only need integer arithmetic and logic operations, they are straightforwardly executed in the already existing ALU, also allowing a faster emulation of Posits
- Being able to write functions as a sequence of arithmetic-logic operations allows us to vectorize them exploiting already existing SIMD (Single Instruction–Multiple Data) engines.

In particular, in this extension, we also propose a new fast and approximated version of the Extended Linear Unit (ELU) activation function.

Moreover, if we consider really low-power devices that do not embed a floating point unit but only an arithmetic logic unit, the approach proposed can become very interesting to enable DNN processing even in this class of devices (although for inference only, not for training).

Furthermore, we investigate operator tabulation as a different approach to speed up Posit emulation without constraints on the exponent configuration. This allows us to accelerate basic arithmetic operators like sum and multiplication that are not suitable for being implemented as L1 functions. Although very powerful, this approach has clear limitations to its scalability, having a considerable spatial complexity.

### Paper Structure

The paper is organized as follow: Section 2 introduces the Posit format, proposing novel approaches to approximation and speed-up of Posit arithmetic, exploiting the 0-bit exponent Posit configuration. Section 3 describes the cppPosit library implemented in Pisa for the computation of the new numerical format. Section 4 introduces the hyperbolic tangent and ELU activation functions along with their approximations. Section 5 shows the results of our approach with DNN and common benchmarking datasets. Finally, Section 6 provides the conclusions.

## 2. Posit Arithmetic

**Figure 1:**
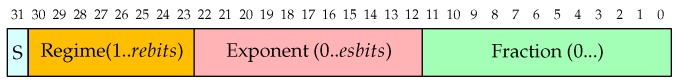
Illustration of the of 32-bit Posit data type.

The Posit format has been introduced by John L. Gustafson in Reference [8] and was further investigated in Reference [9,10,12]. The format is a fixed-length one with up to 4 fields as also reported in Figure 1:

- Sign field: 1-bit
- Regime field: variable length, composed of a string of bits equal to 1 or 0 ended respectively by a 0 or 1 bit.
- Exponent field: at most *es* bits
- Fraction field: variable length mantissa

Given a Posit on nbits;esbits, represented by the integer *X*, and *e* and *f* respectively as the exponent and fraction values, the real number *r* represented by that encoding is as follows:

$$r=\left\{\begin{array}{c} 0,\;\mathrm{if}\;X=0\\ \mathrm{NaN},\;\mathrm{if}\;X=-2^{\left(nbits-1\right)}\\ sign\left(X\right)\times useed^{k}\cdot 2^{e}\cdot \left(1+f\right),\;\mathrm{otherwise} \end{array}\right.$$

**Figure 2:**
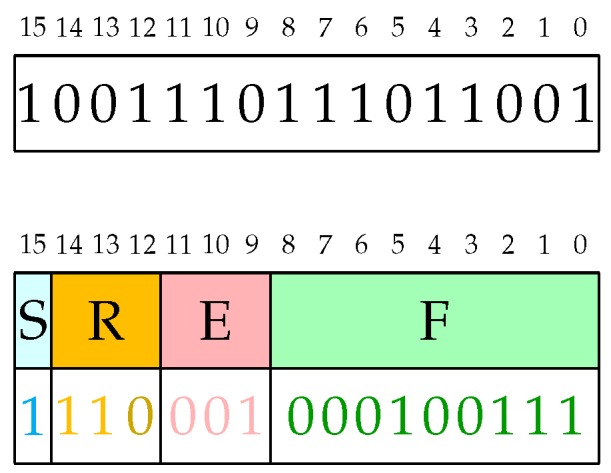
An example of a 16-bit Posit with 3 bits for the exponent (esbits = 3): Given the sequence on top of the figure, after detecting that it starts with one 1, we have to compute the 2’s complement of all the remaining bits (passing from 001-110-111011001 to 110-001-000100111). Then, we can proceed to decode the Posit. The associated real value is therefore 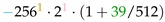. The final value is therefore −512·(1+39/512)=−551 (exact value, i.e., no rounding, for this case).

An example of Posit decoding operation is shown in Figure 2.

The design of a hardware Posit Processing Unit (PPU) as a replacement for the FPU has already started on several universities worldwide, but it will take time for their availability on real platforms. Fortunately, we can still do many things related to DNNs even in the absence of a hardware PPU. Furthermore, when DNN weights can be represented with less than 14-bit Posits, we can tabulate some core operations like sum and multiplication (see Section 3.1) and can use the ALU for other operations that will be shown hereafter in order to reduce the number of tables.

As reported above, the process of decoding a Posit involves the following steps: obtaining regime value by reconstructing the bit-string, building exponent, and extracting fraction. We can make use of C low-level building blocks to speed up the decoding:

- Count leading zeros: using the embedded __builtin_clz C function that several CPU families provide in hardware [13].
- Next power of two: used to extract the fraction. An efficient way to obtain the next power of two, given a representation *X* on 32 bit, is the following:
      next_p2(X) -> Y
          Y = X - 1
          Y = Y | X >> 1
          Y = Y | X >> 2
          Y = Y | X >> 4
          Y = y | X >> 8
          Y = Y | X >> 16
          Y = Y + 1
  This approach copies the highest set bit to all the lower bits. Adding one to such a string will result in a sequence of carries that will set all the bits from the highest set to the least significant one to 0 and the next (in order of significancy) bit of the highest set to 1, thus producing the next power of two. Let us use an example. Suppose $X={\left(5\right)}_{10}={\left(0101\right)}_{2}$. At the first step, $Y={\left(0100\right)}_{2}$. At the second step, $Y={\left(0100\right)}_{2}{|\left(0010\right)}_{2}={\left(0110\right)}_{2}$. At the next step, $Y={\left(0110\right)}_{2}{|\left(0001\right)}_{2}={\left(0111\right)}_{2}$. From now on, *Y* will remain set to $Y={\left(0111\right)}_{2}$. At the last step, $Y={\left(0111\right)}_{2}+{\left(0001\right)}_{2}={\left(1000\right)}_{2}={\left(8\right)}_{10}$, that is the next power of two starting from 5.

### 2.1. The Case of No Exponent Bits (esbits = 0)

When using a Posit configuration with zero exponent bits (*esbits* = 0), some interesting properties arise. In this case, we can express the real number represented by the Posit as follows:

(1) $$x=2^{k}\cdot \left(1+\phi \cdot 2^{-F}\right)$$

where ϕ is the fraction field and *F* the fraction length. The value of *k* depends on the regime length R. In particular, k=−R for x<1 (from now on $x^{-}$) and k=R−1 for x>=1 (from now on $x^{+}$). If we denote the bit immediately following the regime bit string (stop-bit) as σ, we can express the value of *R* as $R=N-2-\sigma_{p}$, where $\sigma_{p}$ is the position of the stop-bit in the Posit bit-string. For $x^{-}$, we can note that, substituting the expression for F=N−2−R in (1), we get the following expression:

(2) $$x^{-}=2^{-R}+\phi \cdot 2^{-(N-2)}=2^{N-2}\cdot \left(2^{-F}+\phi \right)$$

Moreover, we can link $x^{-}$ with its representation *X* using Equation (3), obtaining:

(3) $$x^{-}=X\cdot 2^{-(N-2)}$$

**Figure 3:**
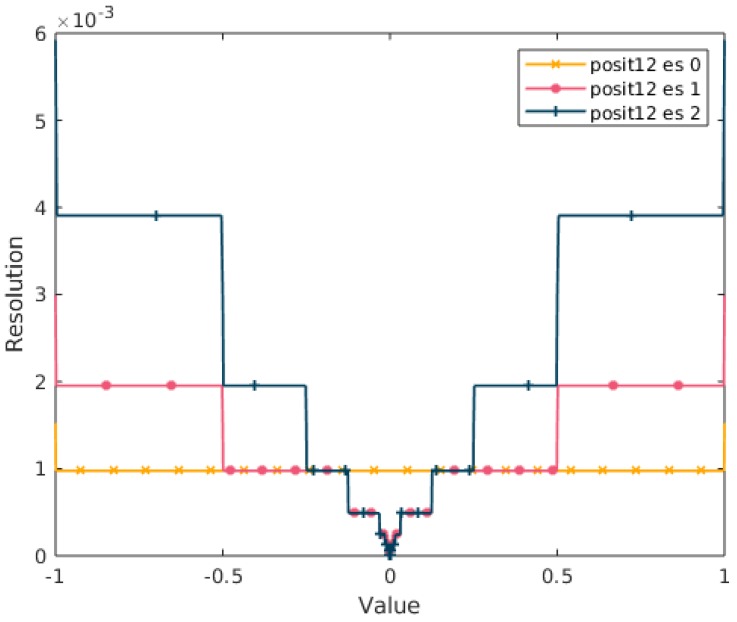
Resolution of a 12-bit Posit when varying the exponent size. With a 0-bit exponent, the Posit resolution in the [0,1] range is the one of a 12-bit fixed point format.

A particular property emerges with 0-bit exponent Posits when considering the [0,1] range. In fact, if we plot the resolution (that is the decimal difference between the real numbers represented by two consecutive bit-strings) of a Posit$\langle X,0\rangle$ in [0,1] we obtain the resolution of a fixed-point format. This property is visualized in Figure 3. This is a very important property that will be exploited below.

As we will see below, the novel equations introduced above for the first time play an important role for deriving fast approximation of activation functions in DNNs. Equation (3) says also that a Posit with zero exponent bits can be interpreted as a fixed point number with a shift of (N−2) bits. This has implications on the accuracy and further operations.

An example of how to exploit the expressions discovered in the previous section is building a fast approximated inversion operator. Given *x*, we want to find a fast and efficient way to compute *y* such that x·y≈1. In the following, we will consider only positive values of x. The simplest case is when f=0. Let us consider x>1; we simply need to apply a reduction of the regime length by 1 as in Equation (4).

(4) $$x\cdot y=2^{R_{x}-1-R_{y}}=1\overset{}{\to }R_{y}=R_{x}-1$$

A trickier case is when f>0. Here, we can easily see that $k_{x}+1=k_{y}$, that implies $R_{x}=R_{y}$. Therefore, we get Equation (5).

(5) $$x\cdot y=2^{-1}\cdot \left(1+f_{x}\cdot f_{y}\cdot 2^{-2F_{x}}+\left(f_{x}+f_{y}\right)\cdot 2^{-F_{x}}\right)=1$$

Then, discarding the term $f_{x}\cdot f_{y}\cdot 2^{-2F_{x}}$, we obtain Equation (6):

(6) $$1+\left(f_{x}+f_{y}\right)\cdot 2^{-F_{x}}=2\overset{}{\to }f_{y}=2^{F_{x}}-f_{x}$$

The latter can be obtained by simply bitwising-not $f_{x}$ and by adding 1, thus obtaining Equation (7):

(7) Y=X⊕(¬signmask)

where ⊕ is the exclusive or (XOR) operator, ¬ is the bitwise negation operator, and *signmask* is the a mask obtained as shown in the following pseudo-code. For example, given a 5-bit Posit, the signmask is simply ${\left(10000\right)}_{2}$. The pseudocode also takes into account the holder size; in fact, a 5-bit Posit may be held by an 8-bit integer. This means that, for this holder type, the signmask produced by the pseudocode is ${\left(11110000\right)}_{2}$.

A pseudo-code implementation for f>0 (otherwise, we simply invert the sign) is as follows:

inv(x) -> y

X = x.v     // ’v’ field: bit-string representing the Posit

msb = 1 << (N-1)

signmask = ~((msb | msb -1) >> 1)

Y = X ^ (~signmask)    // negation operator followed by XOR operator (C-style)

y(Y)

Another useful function to implement as bit manipulation is the one’s complement operator (8)

(8) y=1−x

This is of interest when x∈[0,1]. In this case, y∈[0,1], of course. From Equations (1) and (2), we can rewrite the operator as in Equation (9).

(9) $$y=1-2^{k}\cdot \left(1+\phi \cdot 2^{-F}\right)=1-2^{N-2}\cdot \left(2^{-F}+\phi \right)$$

Since we can link *x* to its representation *X*, we obtain (10).

(10) $$y=1-X\cdot 2^{-(N-2)}$$

Then, we can also link *y* to *Y*, obtaining (11).

(11) $$Y=2^{N-2}\cdot \left[1-X\cdot 2^{-(N-2)}\right]=2^{N-2}-X$$

The latter can be obtained easily with an integer subtraction only using the ALU. A pseudo-code implementation is the following:

comp_one(x) -> y

X = x.v    // ’v’ field: bit-string representing the Posit

invert_bit = 1 << (N-2)

Y = invert_bit - X

y(Y)

when esbits=0, we know that $x=2^{k}\cdot \left(1+\phi \cdot 2^{-F}\right)$. when doubling/halving *x*, we simply increment/decrement the exponent *k* by 1. For 0-bit exponent Posits, this operation corresponds to one left shift for doubling and one right shift for halving the number. For instance, let us take a Posit$\langle 5,0\rangle$ with the value 3/4. The correspondent bit-string will be ${\left(00110\right)}_{2}$. If we shift it by one position right, we will get ${\left(00011\right)}_{2}$, that is the bit-string corresponding to a Posit with value 3/8.

### 2.2. FastSigmoid

**Figure 4:**
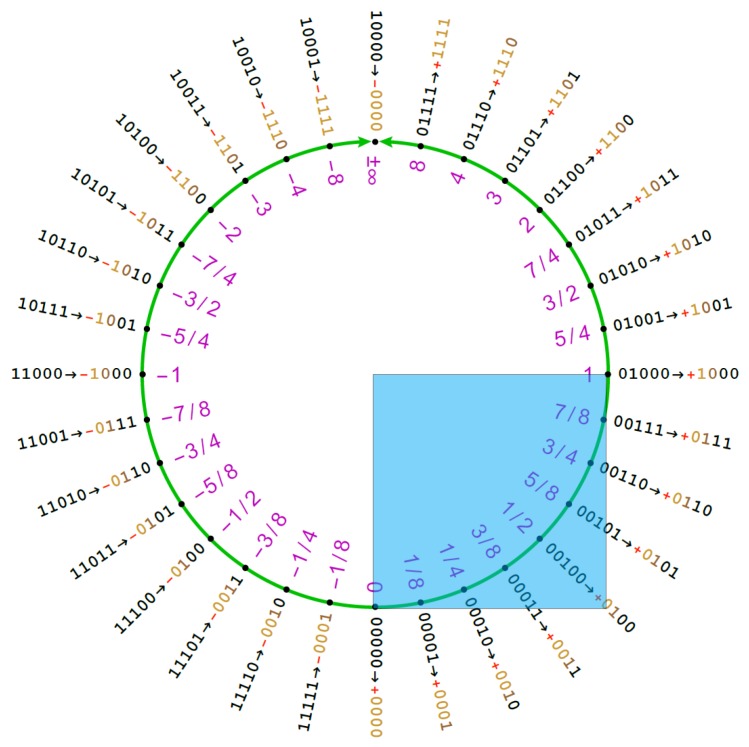
The [0,1] quadrant in the Posit ring.

As pointed out in Reference [8], if we plot the 2’s complement value of the signed integer representing the Posit against the real number obtained from Equation (2), we obtain an S-shaped function very similar to the sigmoid curve. What we need to do is to rescale it to have the co-domain ∈[0,1] and to shift it in order to center it in 0. To bring the Posit in [0,1], we must notice that the quadrant is characterized by having the two most significant bits set at 00 (see Figure 4).

Moreover, we can notice that adding the *invert bit* seen in previous sections to the Posit representation means moving it a quarter of the quadrant. In fact with esbits=0, when adding the invert bit, we are adding $2^{N-2}$, that is equal to $L=\frac{1}{minpos}$, which is the number of Posits that fit in a single quarter of a ring. This means moving *L* times along the Posit ring, thus skipping a quarter of it. A pseudo-code implementation of this transformation is the following:

fastSigmoid(x) -> y

X = x.v     // ’v’ field: bit-string representing the Posit

Y = (invert_bit + (X >> 1)) >> 1

y(Y)

**Figure 5:**
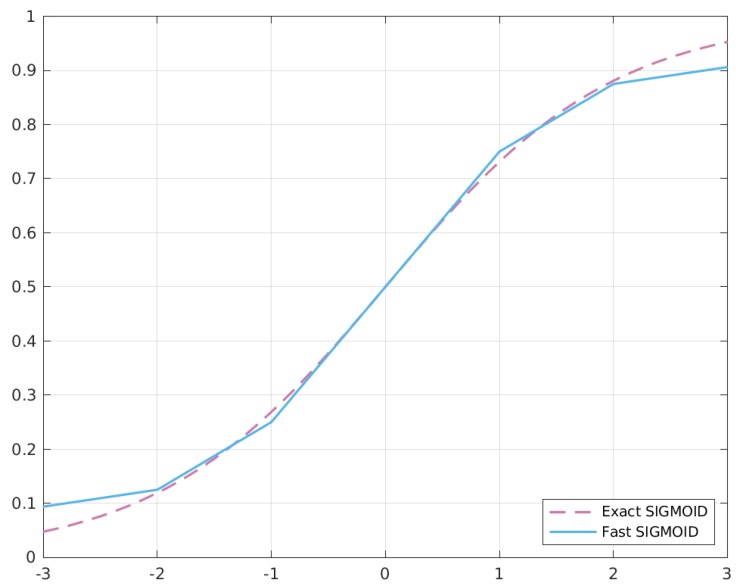
Accuracy comparison between the exact and approximated versions of the Sigmoid function.

In order to understand how this code works, we need to separate the analysis for $x^{-}$ and $x^{+}$, considering only positive values, since the reasoning is symmetric for negative ones. Figure 5 shows the behaviour of the two sigmoid versions.

We know that, for values of x∈[0,1], the behaviour of *x* is like the one of a fixed point representation, so the first right shift is simply a division by two. When we add the *invert bit*, we move the Posit in the northeast ring quarter ([1,+NaR)). After this addition, the last shift can be considered as a division by two as well, thus obtaining the following:

(12) $$y=\frac{x}{4}+\frac{1}{2}$$

Equation (12) is also the first-order Taylor expansion of the Sigmoid function in $x_{0}=0$.

With *x* represented as the bit-string $X=(0,1\left[R_{x}\right],0,\phi_{x})$, the right shift will produce $X^{\prime }=\left(0,0,1\left[R_{x}-1\right],0,\phi_{x}^{\prime }\right)$. Now, with some computation, we can express $x^{\prime }$ as a function of *x* and $R_{x}$, obtaining (13).

(13) $$\frac{x}{2^{2\cdot R_{x}}}+\frac{2^{2\cdot R_{x}}-3\cdot 2^{R_{x}-1}}{2^{2\cdot R_{x}}}$$

when adding the *invert bit*, we obtain $X^{\prime \prime }=\left(0,1\left[R_{x}+1\right],0,\phi_{x}\right)$. Finally, with the last right shift, we obtain (14).

(14) $$\frac{x}{2^{2\cdot R_{x}+1}}+3\cdot \frac{2^{2\cdot R_{x}}-2^{R_{x}}}{4\cdot 2^{2\cdot R_{x}}}$$

We know that we can approximate $R_{x}∼\log_{2}\left(x\right)\overset{}{\to }x∼2^{R_{x}}$. If we substitute it back in Equation (14), we obtain Equation (15), close to $sigmoid(R_{x})$:

(15) $$\frac{3\cdot 2^{R_{x}}-1}{4\cdot 2^{R_{x}}}$$

## 3. CppPosit Library

For this paper, we employ our software implementation of Posit numbers developed at the University of Pisa, called cppPosit. As already described in References [9,12], the library classifies Posit operations into four different classes (from L1 to L4), with increasing computational complexity.

Among the others, L1 operations are the ones we want to focus on, since they can be fully emulated with an ALU. For this reason, they provide means to produce very efficient operators, as reported in Table 1.

This level supports Posit reciprocation and sign-negation as well as one’s complement. Furthermore, when dealing with 0 exponent-bit configuration, they provide the fast and approximated sigmoid function (FastSigmoid) as described in Reference [8] and the fast approximation of the hyperbolic tangent (FastTanh) investigated in Reference [12]. Other interesting operators that require 0 exponent bits are the double and half functions. It is clear that, given these requirements, it is not always easy to derive a simple expression for a particular function that can be implemented in an L1 way. However, the effort put in this step is completely rewarded since it brings both faster execution both in a emulated and hardware Posit Processing Unit (PPU) and reduction of transistor occupation when dealing with hardware implementation of the unit.

### 3.1. Tabulated Posits

In the absence of proper hardware support of a Posit Processing Unit (PPU), there still is the need for speeding up the computation. An interesting mean to cope with this problem is the pre-computation of some useful Posit operators in look-up tables. These lookup tables (LUTs) become useful when the number of bits is low (e.g., nbits<12). The core idea is to generate tables for the most important arithmetic operations (addition/subtraction and multiplication/division) for all combinations of a given Posit configuration nbits,esbits. Moreover, some interesting functions can be tabulated in order to speed up their computation, like *logarithm* or *exponentiation*. Given an nbits bit Posit with a naive approach, a table will be $T\in P^{R\times C}$ where $R=C=2^{nbits}-1$.

Depending on the underlying storage type T, each table entry will occupy b=sizeof(T) bits. Typically, there will be between N=8 and N=10 tables for a Posit configuration. This means that the overall space occupation will be S=N·(R·C)·b.

Table 2 shows different per-table occupations of different Posit configurations. As reported, only Posits with 8 and 10 bits have reasonable occupation, considering current generation of CPUs. In fact, we can obtain a considerable speed-up when one or more tables can be entirely contained inside the cache.

In order to reduce both LUT size and their number, we can exploit some arithmetic properties:

- Addition and subtraction are respectively symmetric and antisymmetric. The two tables can be merged into one, and only one half of it is required (above or below the main diagonal).
- Multiplication and division can be simplified through logarithm properties. Given p=x·y, we can apply log operator on both sides (see Reference [14]), thus obtaining log(p)=log(x·y). From logarithm properties, this results in log(p)=log(x)+log(y) Finally, going back with exponentiation, we get $p=e^{\log(x)+\log(y)}$. Since tabulation of single operators scales linearly with the Posit size, it is feasible only to store exp,log instead of multiplication and division, thus exploiting addition/subtraction LUT for the computation.
- We can compact multiplication tables even more by exploiting the fast inversion (L1) shown in Section 2. Suppose to have two Posit numbers x,y and their reciprocates, if we want to provide every multiplication or division combination, we would build a LUT like in Table 3. This table would result in 16 entries for only 4 numbers, hence not manageably growing with Posit size. If we apply the L1 inversion and symmetry of negative values, we only need to store the operations for x·y and x/y, thus resulting in a LUT size of only 2 elements for the same amount of numbers, as shown in Table 4.

### 3.2. Type Proxying

When dealing with Posit configuration with esbits≠0 it is not possible to exploit fast approximation of operators that relies on this property. A possible solution is to switch to a different Posit configuration with 0 exponent bits and higher total number of bits to exploit a fast approximation and to then switch back to the original one.

Increasing the number of bits is also useful when the starting Posit configuration has already 0 exponent bits. In fact, increasing nbits for the operator computation increases the accuracy of the computation, avoiding type overflows.

Given a Posit configuration $P1\langle X,Y\rangle$, the basic idea is to proxy through a configuration $P2\langle Z,0\rangle$ with Z≫X. The core step in the approach is the Posit conversion between different configurations. The base case is converting $P1\langle X,0,T1\rangle \overset{}{\to }P2\langle Z,0,T2\rangle$, with Z≫X and sizeof(T2)≫sizeof(T1). In this case, the conversion operation is the following:

convert0(p1) -> p2

v1 = p1.v     // ’v’ field: bit-string representing the Posit

v2 = cast<T2>(v1) << (Z - X)

p2.v2 = v2

### 3.3. Brain Posits

The idea behind Brain Floats is to define a Float16 with the same number of bits for the exponents of an IEEE 754 Float32. BFloat16 is thus different from IEEE 754 Float16, and the rationale of its introduction is that, when we have a DNN already trained with IEEE Float32, we can perform the inference with a BFloat16 and we can expect a reduced impact on the accuracy due to the fact that the dynamic range of a BFloat16 is the same as that of IEEE Float32. Following the very same approach, we can define *Brain Posits* to be associated to the Posit16 and Posit32 that will be standardized soon. In particular, BPosit16 can be designed in such a way that it has the same dynamic range of a standard Posit32, which will be the one with 2 bits of exponent. Since we are using the Posit format, we can define the BPosit16 as the 16-bit Posit having a number of bits for the exponent such that its dynamic range is similar to the one of Posit<32,2>. Using the same approach, we will define BPosit8, where the number of bits for the exponent, in this case, must be the one that allows the BPosit8 to cover most of the dynamic range of the standard 16-bit Posit, which is the Posit<16,1>. In the following, we will perform some computations to derive the two number of exponents. Indeed, another interesting aspect of type proxying is that we can also reduce the total number of bits while increasing the exponent ones and still being able to accommodate the entire dynamic range. In doing so, we need to know the minimum number of exponent bits of the destination type. Suppose we are converting from Posit $P1\langle X_{1},Y_{1}\rangle$ to Posit $P2\langle X_{2},Y_{2}\rangle$, with $X_{1}>X_{2}$. We know that the maximum value for $P_{1}$ (similarly, it holds for $P_{2}$ as well) is $max_{1}={\left[2^{2^{Y_{1}}}\right]}^{X_{1}-2}$ If we set the inequality $max_{2}\geq max_{1}$ and we apply logarithms to both sides, we get $\left(X_{2}-2\right)\cdot 2^{Y_{2}}\geq \left(X_{1}-2\right)\cdot 2^{Y_{1}}$ From this, we obtain the rule for determining the exponent bits of the destination type:

(16) $$Y_{2}\geq \log_{2}\left(\frac{X_{1}-2}{X_{2}-2}\right)+Y_{1}$$

From Equation (16), we can derive some interesting cases. A Posit $P1\langle 16,1\rangle$ can be transformed into a Posit $P2\langle 8,2\rangle$ without a significant loss in the dynamic range. Furthermore, the same holds for a Posit $P1\langle 32,2\rangle$, which can be approximated using Posit $P1\langle 16,3\rangle$.

For all this reasons, the Brain Posits proposed in Table 5 might deserve a hardware implementation too.

## 4. Hyperbolic Tangent, Extended Linear Unit, and their Approximations

**Figure 6:**
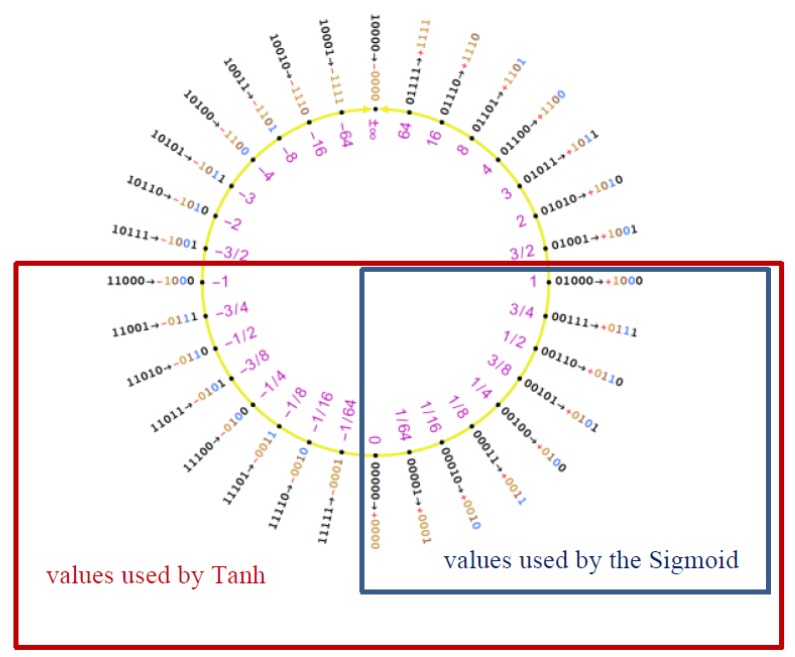
Five-bit Posit mapping to the Posit circle: As reported, the tanh function manages to cover the lower half of the circle while the sigmoid one covers only the quarter [0,1].

The hyperbolic tangent (*tanh* from now on) is a commonly used activation function. Its use over the sigmoid function is interesting since it extends the sigmoid codomain to the interval [−1,1]. This allows both the dynamic range of the sigmoid in the output to be exploited twice and the negative values in classification layers during training to be given meaning. The first advantage is particularly important when applied to Posit, especially to small-sized ones. In fact, when considering the sigmoid function, if we apply it to a Posit$\langle X,Z\rangle$, we practically obtain in the output the dynamic range of a Posit$\langle X/2,Z\rangle$, that is, for instance, quite limiting for Posits with 8 to 14 number of bits. Figure 6 stresses this point, highlighting how the tanh function insists on the two most dense quarters of the Posit circle (the interval [−1,1] occupies half of the Posit circle).

However, the sigmoid function has an important property, as shown in Table 1 and in Reference [8]: it can be implemented as L1 function, thus having a fast and efficient approximation only using integer arithmetics. The idea is to use the sigmoid function as a building block for other activation functions, only using a combination of L1 operators. We know that the sigmoid function is:

(17) $$sigmoid\left(x\right)=\frac{1}{e^{-x}+1}$$

Now, we can scale and translate (17) to cover the desired range [−1,1] on the output obtaining the scaled sigmoid:

(18) $$sSigmoid_{k}\left(x\right)=k\cdot sigmoid\left(k\cdot x\right)-k/2$$

Equation (18) is useful when setting k=2, thus obtaining the tanh expression in (19):

(19) $$sSigmoid_{2}\left(x\right)=\left(e^{2x}-1\right)/\left(e^{2x}+1\right)=tanh\left(x\right)=2\cdot sigmoid\left(2\cdot x\right)-1$$

From this formulation, we want to build an equivalent one that only uses L1 operators to build the approximated hyperbolic tangent, switching from sigmoid to the fast approximated version called FastSigmoid. Since we are dealing with 0 exponent bit Posits, the operations of doubling the Posit argument, computing the FastSigmoid, and doubling again is just a matter of bit manipulations, thus efficiently computed. However, the last step of subtracting 1 to the previous result is not an L1 operator out-of-the-box; thus, we reformulate the initial expression obtaining (20):

(20) $$tanh\left(x\right)=-\left(1-2\cdot sigmoid(2\cdot x)\right)$$

If we consider only negative arguments *x*, we know that the result of the expression 2·sigmoid(2·x)) is always in the unitary region. This, combining with the 0 exponent bit hypothesis allows us to implement the inner expression with the 1’s complement L1 operator seen in Table 1. The last negation is obviously an L1 operator; thus, we have the L1 fast approximation of the hyperbolic tangent in (21):

(21) FastTanh(x)=−(1−2·FastSigmoid(2·x))

Finally thanks to the antisymmetry of the tanh function, we can extend what we have done before to positive values. The following is a pseudo-code implementation:

FastTanh(x) -> y

x_n = x > 0 ? -x:x

s = x > 0

y_n = neg(compl1(twice(FastSigmoid(twice(x_n)))))

y = s > 0 ? -y_n:y_n

As already described, tanh and sigmoid functions can be implemented in their fast approximated version. However, the use of such kinds of shapes presents the well-known behaviour of vanishing gradients [15]; for this reason, ReLU -like functions (e.g., ELU, Leaky-ReLU, and others) are preferable when dealing with a large number of layers in neural networks. As in Reference [15], the ReLU activation function is defined as in (22):

(22) $$ReLU\left(x\right)=\left\{\begin{array}{c} 0,\;\mathrm{if}\;x\leq 0\\ x\;\mathrm{otherwhise} \end{array}\right.$$

Its use is important in solving the vanishing gradient problem, having a non-flat shape towards positive infinity. However, when used with Posit numbers, this function can only cover [0,inf), ignoring the very dense region [−1,0].

In order to provide a more covering function with similar properties, we switch to the Extended Linear Unit (ELU) (23):

(23) $$ELU\left(x\right)=\left\{\begin{array}{c} \alpha \cdot \left(e^{x}-1\right),\;\mathrm{if}\;x\leq 0\\ x\;\mathrm{otherwhise} \end{array}\right.$$

This function is particularly interesting when α=1 (24), covering the missing dense region from the ReLU one:

(24) $$ELU\left(x\right)=\left\{\begin{array}{c} e^{x}-1,\;\mathrm{if}\;x\leq 0\\ x\;\mathrm{otherwhise} \end{array}\right.$$

**Figure 7:**
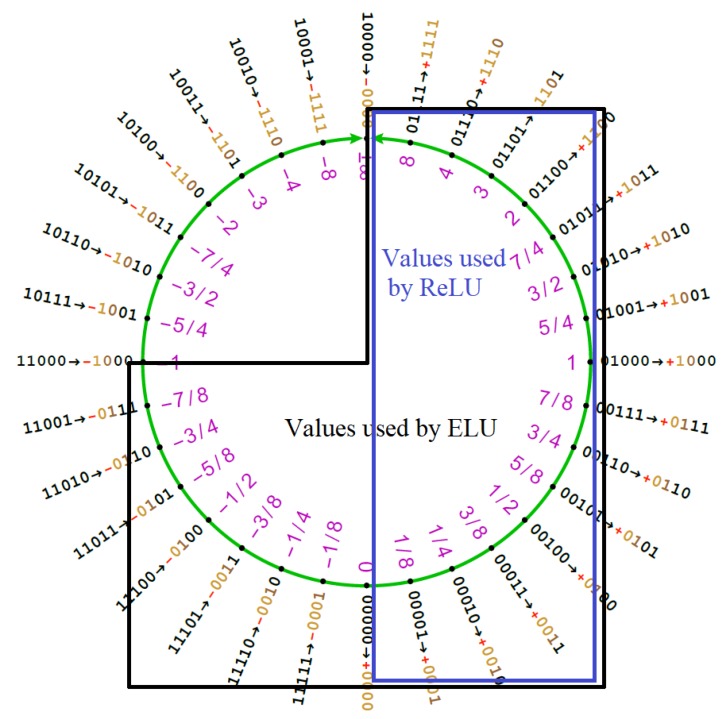
The Posit circle when the total number of bits is 5: The extended linear unit uses all the numbers in [−1,inf), while the ReLU function uses only the ones in [0,inf).

Figure 7 shows the difference in Posit ring region usage of ELU and ReLU functions. It is remarkable how the ELU function manages to cover all the high density regions of the Posit ring. Moreover, the ELU function brings interesting normalization properties across the neural network layers as proven in Reference [16]. This helps in keeping stable the range of variation of the weights of the DNN.

From Equation (24), we can build a L1 approximation exploiting operators in Table 1. The ELU(x) behaviour for x>0 is the identity function, that is L1 for sure. The first step for negative *x* values is seeing that the ELU expression is similar to the reciprocate of Sigmoid function (17). We can manipulate (17) as follows:

(25) $$\begin{array}{c} \mathrm{Sigmoid}\left(-x\right)=\frac{1}{1+e^{x}} \end{array}$$

(26) $$\begin{array}{c} 1/\mathrm{Sigmoid}\left(-x\right)=1+e^{x} \end{array}$$

(27) $$\begin{array}{c} 1/\left(2\cdot \mathrm{Sigmoid}\left(-x\right)\right)=\frac{1+e^{x}}{2} \end{array}$$

(28) $$\begin{array}{c} 1/\left(2\cdot \mathrm{Sigmoid}\left(-x\right)\right)-1=\frac{1+e^{x}}{2}-1=\frac{e^{x}-1}{2} \end{array}$$

(29) $$\begin{array}{c} 2\cdot \left[1/\left(2\cdot \mathrm{Sigmoid}\left(-x\right)\right)-1\right]=e^{x}-1 \end{array}$$

We need to prove that the steps involved are L1 operations. The step in Equation (25) is always L1 for esbits=0 thanks to fast Sigmoid approximation. The result of this step is always on [1,2]. The step in Equation (26) is always L1, and the output is on [1/2,1]∈[0,1]. The step in Equation (27) is always L1 for esbits=0, and the output is on [0,1/2]. The step in Equation (28) is L1 since the previous step output is in the unitary range [0,1]. The output of this step is in [0,1] as well. Finally, the last step is L1 for esbits=0. Expression (29) is exactly the ELU expression for negative values of the argument.

A pseudo-code implementation of the FastELU using only L1 operations is shown below:

FastELU(x) -> y

y_n = neg(twice(compl1(half(reciprocate(FastSigmoid(neg(x)))))))

y = x > 0 ? x:y_n

**Figure 8:**
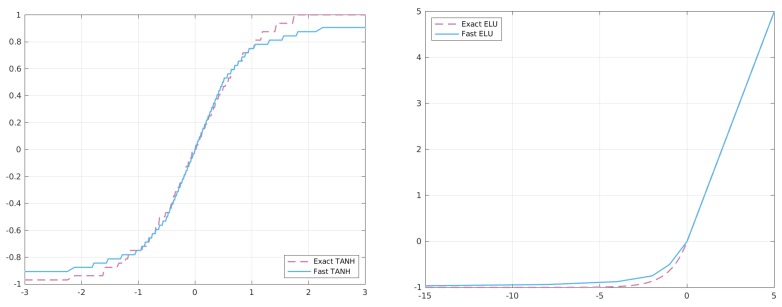
Comparison between the exact and approximated versions of hyperbolic tangent (TANH) and extended linear unit (ELU).

Figure 8 shows the behaviour of the two functions when approximated with our approach.

## 5. Implementation Results

**Figure 9:**
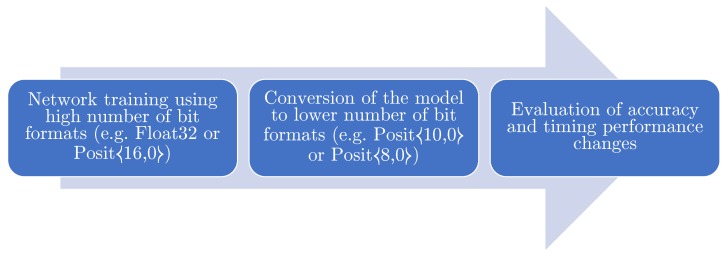
Flowchart for the proposed method: models are trained using formats with high bit count like Float32 or, in the future, Posit$\langle 16,0\rangle$. The models obtained this way are then converted to formats with lower bit count (e.g., Posit$\langle 8,0\rangle$) to increase space efficiency and bandwidth.

In this section, the different proposed activation function performances are analyzed in both the exact and approximated fashions when used as activation function in the LeNet-5 neural network model [17]. As shown in Figure 9, the neural network is trained with the MNIST digit recognition benchmark (GTRSB) [17] and the German Traffic Road Sign Benchmark [18] datasets using the Float32 type. The performance metrics involved are the testing accuracy on said datasets and the mean sample inference time. Testing phase is executed converting the model to $Posit\langle X,Y\rangle$ type and to SoftFloat32 (a software implementation of floats). We used SoftFloats in order to ensure a fair comparison between the two software implementations due to the absence of proper hardware support for Posit type.

Benchmarks are executed on a 7th generation Intel i7-7560U processor, running Ubuntu Linux 18.04, equipped with GCC 8.3. Benchmark data is publicly available in References [17]. The C++ source code can be downloaded from Reference [19].

As reported in Table 6 and Table 7, the approximated hyperbolic tangent can replace the exact one, with a small degradation in accuracy but improving the inference time of about 2 ms in each Posit configuration. Moreover, the performance of FastTanh also overcome FastSigmoid in terms of accuracy. Furthermore, as reported in Table 8 and Table 9, the approximated ELU function can replace the exact one, with little-to-no accuracy degradation, improving the inference time of about 1 ms in each Posit configuration. Moreover, performance of FastELU also overcomes the ReLU in terms of accuracy, showing the benefits of covering the additional region in [−1,0]. At the same time, the FastELU is not much slower than ReLU, thus being an interesting replacement to increase accuracy of Posits with few bits (e.g., Posit$\langle 8,0\rangle$) without losing too much in time complexity.

If we compare FastELU and FastTanh, their performance are quite similar in the benchmarks provided. However as already said in Section 4, increasing the number of layers in the neural network model can lead to the so called “vanishing gradient” problem; s-shaped functions like sigmoid and hyperbolic tangent are prone to this phenomenon. This has been proven not to hold for ReLU-like functions.

The results highlight how Posits from $\mathrm{Posit}\langle 16,0\rangle$ to $\mathrm{Posit}\langle 10,0\rangle$ are a perfect replacement for float numbers; $\mathrm{Posit}\langle 10,0\rangle$ is a particularly interesting format since it offers the best data compression without any drop in accuracy. This reasonably makes $\mathrm{Posit}\langle 10,0\rangle$ the configuration of choice for low-precision inference when using Posits.

## 6. Conclusions and Future Work

In this work, we have introduced some interesting properties of Posit format for the specific configuration having zero exponent bits (esbit=0), that allows building fast arithmetic operators that only requires ALU support. In particular, we have derived two novel fast approximated versions of two important activation functions in neural networks: the hyperbolic tangent and the extended linear unit. These approximations are fast since they involve only bit manipulations (at the so-called “L1 level”). This means that such functions do not need to be implemented in hardware within the so-called Posit processing unit. Instead, they can be efficiently computed using the ALUs of most of the current CPUs. We have used this approximation to speed up the inference phase of deep neural networks. The proposed approximations have been tested on common deep neural network benchmarks. The use of this approximations resulted in a slightly less accurate neural network with respect to the use of the (slower) exact version but with better performance in terms of mean sample inference time of the network. In our experiment, the FastTanh and FastELU functions also outperform both the ReLu and the FastSigmoid (a well-known approximation of the sigmoid function), a de facto standard activation function in neural networks. Future developments of the work will include porting the Posit format inside the Apollo Autonomous Driving Framework to test it on the assisted/autonomous driving scenario; this will allow us to test our approach in object detection and semantic segmentation tasks. We plan to implement a Field Programmable Gate Array (FPGA) based Posit Processing Unit (PPU) in order to evaluate real-world hardware performance of our library. Furthermore, we are actively working to port the cppPosit library for the new RISC-V processor architecture; we plan to develop both a software-accelerated version using the vector extension of the RISC-V Instruction Set Architecture (ISA) and an intellectual property (IP) core for the RISC-V hardware architecture. 

## Figures and Tables

**Table 1:** Most interesting L1 operators implemented in cppPosit and their requirements to be applied on the argument *x*.

Operation	Approximated	Requirements
2·x	no	*esbits = 0*
x/2	no	*esbits = 0*
1/x	yes	*esbits = 0*
1−x	no	*esbits = 0, x∈[0,1]*
FastSigmoid(*x*)	yes	*esbits = 0*
FastTanh(*x*)	yes	*esbits = 0*
FastELU(*x*)	yes	*esbits = 0*

**Table 2:** Table occupation for various configurations.

Total Bits (X)	Storage Type Bits (b)	Per-Table Occupation
**8**	8	64 KB
**10**	16	2 MB
**12**	16	32 MB
**14**	16	512 MB
**16**	16	8 GB

**Table 3:** All the possible combinations for multiplying and dividing two Posit numbers.

	1/x	x	−1/x	−x
**1/y**	1/xy	x/y	−1/xy	−x/y
**y**	y/x	xy	−y/x	−xy
**−1/y**	−1/xy	−x/y	1/xy	−x/y
**−y**	−y/x	−xy	−y/x	xy

**Table 4:** All the possible combinations for multiplying and dividing two Posit numbers: all the cells in italics correspond to the same LUT entry, and all the remaining ones correspond to another LUT entry.

	1/x	x	−1/x	−x
**1/y**	*1/xy*	x/y	*−1/xy*	−x/y
**y**	y/x	*xy*	−y/x	*−xy*
**−1/y**	*−1/xy*	−x/y	*1/xy*	−x/y
**−y**	−y/x	*−xy*	−y/x	*xy*

**Table 5:** Brain Posits.

Standard Posits	Brain Posits
Posit<16,1>	Posit<8,2>
Posit<32,2>	Posit<16,3>
Posit<64,3>	Posit<32,4>

**Table 6:** Comparison using Posits for the MNIST dataset for three different activation functions: fast approximated version of Tanh (FastTanh), exact Tanh, and FastSigmoid. Accuracy of the neural network and mean sample inference time are reported.

Activation	FastTanh (This Paper)		Tanh		FastSigmoid	
	Acc. (%)	Time (ms)	%	ms	%	ms
SoftFloat32	-	-	99.4	8.3	97.1	-
Posit$\langle 16,0\rangle$	99.1	3.2	99.4	5.28	97.1	3.31
Posit$\langle 14,0\rangle$	99.1	2.9	99.4	4.64	97.1	3.09
Posit$\langle 12,0\rangle$	99.1	2.9	99.4	4.66	97.1	3.04
Posit$\langle 10,0\rangle$	99.1	2.9	99.3	4.62	96.9	3.08
bottomrule Posit $\langle 8,0\rangle$	98.6	2.9	98.5	4.84	94.2	3.01

**Table 7:** Comparison using Posits for the GTRSB dataset (see Table 6).

Activation	FastTanh (This Paper)		Tanh		FastSigmoid	
	Acc. (%)	Time (ms)	%	ms	%	ms
SoftFloat32	-	-	94.2	15.2	82.0	-
Posit$\langle 16,0\rangle$	93.5	5.3	93.5	6.2	81.9	5.0
Posit$\langle 14,0\rangle$	93.4	4.2	93.5	5.1	81.9	4.3
Posit$\langle 12,0\rangle$	93.4	4.2	93.4	5.1	81.9	4.3
Posit$\langle 10,0\rangle$	93.4	4.2	93.3	5.1	81.0	4.2
Posit$\langle 8,0\rangle$	93.0	4.0	92.3	5.0	72.1	4.0

**Table 8:** Comparison using Posits for the MNIST dataset for three different activation functions: fast approximated version of ELU (FastELU), exact ELU, and ReLU. Accuracy of the neural network and mean sample inference time are reported.

Activation	FastELU (This Paper)		ELU		ReLU	
	Acc. (%)	Time (ms)	%	ms	%	ms
SoftFloat32	-	-	98.6	8.8	89.1	6.3
Posit$\langle 16,0\rangle$	98.5	3.2	98.6	3.9	89.1	2.0
Posit$\langle 14,0\rangle$	98.5	2.4	98.6	3.1	89.05	2.0
Posit$\langle 12,0\rangle$	98.5	2.3	98.6	3.1	89.0	2.0
Posit$\langle 10,0\rangle$	98.3	2.3	98.5	3.0	89.0	1.9
Posit$\langle 8,0\rangle$	91.1	2.2	90.1	3.0	88.4	1.9

**Table 9:** Comparison using Posits for the GTRSB dataset (see Table 8).

Activation	FastELU (This Paper)		ELU		ReLU	
	Acc. (%)	Time (ms)	%	ms	%	ms
SoftFloat32	-	-	94.2	15.86	92.0	8.2
Posit$\langle 16,0\rangle$	94.0	5.8	94.2	6.37	92.0	5.0
Posit$\langle 14,0\rangle$	94.0	4.6	94.2	5.21	92.0	4.3
Posit$\langle 12,0\rangle$	94.0	4.6	94.2	5.08	92.0	4.3
Posit$\langle 10,0\rangle$	94.0	4.6	94.2	5.0	92.0	4.2
Posit$\langle 8,0\rangle$	92.0	4.6	91.8	5.0	86.8	4.0
